# Diagnostic Criteria and Measurement Techniques of Sarcopenia: A Critical Evaluation of the Up-to-Date Evidence

**DOI:** 10.3390/nu16030436

**Published:** 2024-02-01

**Authors:** Gavriela Voulgaridou, Stefanos Tyrovolas, Paraskevi Detopoulou, Despoina Tsoumana, Mariella Drakaki, Thomas Apostolou, Ioanna P. Chatziprodromidou, Dimitrios Papandreou, Constantinos Giaginis, Sousana K. Papadopoulou

**Affiliations:** 1 Department of Nutritional Sciences and Dietetics, International Hellenic University, 57400 Thessaloniki, Greece; gabivoulg@gmail.com (G.V.); tsoumanadespoina@gmail.com (D.T.); drakakimariella@gmail.com (M.D.); 2 Department of Nutrition and Food Studies, George Mason University, Fairfax, VA 22030, USA; styrovol@gmu.edu; 3 WHOCC Centre for Community Health Services, School of Nursing, The Hong Kong Polytechnic University, Hong Kong SAR, China; 4 Research, Innovation and Teaching Unit, Parc Sanitari Sant Joan de Déu, 08830 Sant Boi de Llobregat, Spain; 5 Instituto de Salud Carlos III, Centro de Investigación Biomédica en Red de Salud Mental, Centro de Investigación Biomédica en Red de Salud Mental (CIBERSAM), 28029 Madrid, Spain; 6 Department of Clinical Nutrition, General Hospital Korgialenio Benakio, Athanassaki 2, 11526 Athens, Greece; 7 Department of Physiotherapy, School of Health Sciences, International Hellenic University, 57400 Thessaloniki, Greece; apostolouthomas@hotmail.com; 8 Department of Public Health, Medical School, University of Patras, 26504 Patra, Greece; ioannachatzi@med.upatras.gr; 9 Department of Clinical Nutrition & Dietetics, College of Health, University of Sharjah, Sharjah P.O. Box 27272, United Arab Emirates; dpapandreou@sharjah.ac.ae; 10 Department of Food Science and Nutrition, School of Environment, University of Aegean, 81400 Myrina, Greece; cgiaginis@yahoo.gr

**Keywords:** sarcopenia, criteria, muscle mass, muscle strength, physical function, physical performance, biomarkers, screening

## Abstract

Sarcopenia, a geriatric syndrome characterized by progressive skeletal muscle mass and function decline, poses a significant health risk among the elderly, contributing to frailty, falls, hospitalization, loss of independence and mortality. The prevalence of sarcopenia varies significantly based on various factors, such as living status, demographics, measurement techniques and diagnostic criteria. Although the overall prevalence is reported at 10% in individuals aged 60 and above, disparities exist across settings, with higher rates in nursing homes and hospitals. Additionally, the differences in prevalence between Asian and non-Asian countries highlight the impact of cultural and ethnic factors, and variations in diagnostic criteria, cut-off values and assessment methods contribute to the observed heterogeneity in reported rates. This review outlines diverse diagnostic criteria and several measurement techniques supporting decision making in clinical practice. Moreover, it facilitates the selection of appropriate tools to assess sarcopenia, emphasizing its multifactorial nature. Various scientific groups, including the European Working Group of Sarcopenia in Older People (EWGSOP), the International Working Group on Sarcopenia (IWGS), the Asian Working Group on Sarcopenia (AWGS), the American Foundation for the National Institutes of Health (FNIH) and the Sarcopenia Definition and Outcomes Consortium (SDOC), have published consensus papers outlining diverse definitions of sarcopenia. The choice of diagnostic criteria should be aligned with the specific objectives of the study or clinical practice, considering the characteristics of the study population and available resources.

## 1. Introduction

Sarcopenia, originating from the Greek “sarx” (flesh) and “penia” (loss), is mostly a geriatric syndrome, characterized by a progressive decrease in skeletal muscle mass and function [1]. It is one of the most common health problems among the elderly. The term sarcopenia was first described as muscle wasting that occurs as a natural procedure of aging [1]. Secondary sarcopenia is considered when other factors related to human chronic diseases are present (beyond aging), such as cancer, heart disease or kidney disease. Evidence suggests that sarcopenia considerably raises the risk of frailty, falls and fall-related injuries, hospitalization, loss of independence and mortality [2]. Old age, physical inactivity, malnutrition and comorbidities have been considered crucial risk factors for sarcopenia [3].

The prevalence of sarcopenia is influenced by several factors, including the living status of individuals (whether they reside in a community, hospital or nursing home), demographic characteristics such as age, ethnicity, genetic background, the specific measurement techniques used for assessing muscle mass and function and the diagnostic criteria applied in clinical evaluation. Notably, living status significantly impacts sarcopenia prevalence, with higher rates observed among nursing home residents (51% in men and 31% in women) and hospitalized individuals (approximately 20%) [4]. This discrepancy is attributed to factors like physical inactivity and malnutrition, which are prevalent in institutionalized patients. Furthermore, sarcopenia appears to be more prevalent in non-Asian countries compared to Asian countries [5].

Muscle wasting resulting from several chronic diseases is known as cachexia. These disorders share the same pathophysiological mechanisms that lead to amino acid mobilization from muscles due to hypercatabolic condition [6]. Importantly, unlike cachexia, sarcopenia does not include a substantial hypercatabolic state, so it involves separate pathways. Sarcopenia is most likely a result of co-morbidities in some patients [7]. Notably, diverse definitions for sarcopenia have been proposed [8]. The prevalence of sarcopenia depends on the definition used and the attributes of the population [8]. This review aims to provide an overview of the used diagnostic criteria for sarcopenia, as well as to elucidate the various measurement techniques utilized for its evaluation.

## 2. Methods

A search was conducted in the PubMed database to identify articles related to the criteria of sarcopenia and measurement techniques. The following terms were utilized: “sarcopenic criteria”, “muscle mass”, “muscle strength”, “physical function”, “measurements” and “sarcopenia assessment”. Synonymous words were also included in the search. The Boolean operator “AND” was used to combine these terms, and “OR” was employed to combine synonymous words.

Additionally, a systematic search on the PubMed database was performed to identify studies comparing the prevalence of sarcopenia using different diagnostic criteria. The applied search string was as follows: (“diagnostic criteria” [tiab] OR “criteria” [tiab]) AND (“Sarcopenia” [Mesh] OR “sarcopenia” [tiab]) AND “compare”. The inclusion criteria focused on health status, specifically incorporating studies with apparently healthy middle-aged and older adults (>50 years old). Studies involving critically ill patients, such as those with cancer, were excluded.

## 3. Diagnosis of Sarcopenia

A multitude of tests and assessment tools have been developed to accurately define sarcopenia [9,10]. The selection of appropriate tests and tools should be contingent upon several factors: (a) the patient’s health status, particularly their mobility, (b) the availability of suitable equipment and (c) the specific purpose of the assessment, including whether it is for screening or follow-up evaluation [9]. Below, we elaborate on the most validated and widely accepted tests and tools utilized for the detection and assessment of sarcopenia.

Previous reviews or meta-analyses have assessed the predictive power of various sarcopenia criteria [11,12]. Most of these studies have not included the recently published definitions of sarcopenia, such as the EWGSOP2, SDOC and AWGS 2 [9,13,14]. Indeed, most recent reviews in the last two years have focused on sarcopenia epidemiology [15], specific diseases [16] or methodological issues of radiological methods [17,18]. To our knowledge, few recent reviews have been published on the predictive validity of sarcopenia definitions in healthy subjects, including all criteria [19] or focusing on specific criteria, such as EWGSOP1 and EWGSOP2 [20]. However, the work of Stuck et al. [19] focused mostly on the clinical outcomes of sarcopenia diagnosis, as derived from different definitions. The originality of the present review lies in the fact that it includes both a critical appraisal of all sarcopenia definitions and a comparative analysis of sarcopenia prevalence with several criteria, and the analysis is not restricted to clinical outcomes.

### 3.1. Case Finding

The strength, assistance with walking, rising from a chair, climbing stairs and falls (SARC-F) questionnaire was first published by Malmström and Morley in 2013 [21]. Its use is also suggested by the EWGSOP2 [9], and it is the most widely used screening test [22]. This questionnaire is a simple, valuable and inexpensive tool and can be used in a variety of clinical settings, including community health care [23]. SARC-F is a self-completed questionnaire, and it includes five items based on the cardinal features of sarcopenia. These five components are strength, assistance with walking, rising from a chair, climbing stairs and falls. The score ranges from 0 to 10, and each item receives 0 to 2 points. Sarcopenia is indicated by a score of 4 out of a total of 10 points [21]. SARC-F, based on both EWGSOP and AWGS diagnostic criteria, shows high specificity but low sensitivity. In a meta-analysis the sensitivity of SARC-F ranged from 27% to 77%, the specificity ranged from 63% to 91%, and the area under the curve (AUC) ranged from 67% to 75% [24]. Nevertheless, it is a valuable tool for detecting individuals with muscle weakness, revealing those who may benefit from sarcopenia treatment [23,25].

A modified version of the SARC-F questionnaire is the SARC-CalF, which is derived from the inclusion of calf circumference (CC). SARC-CalF has higher sensitivity, ranging from 57.4% to 65.5%, as compared to SARC-F, whose sensitivity ranges from 31.5% to 44.8% [26]. Additionally, the diagnostic accuracy of SARC-CalF is higher and ranges from 75.7% to 79.2% compared to SARC-F, whose accuracy ranges from 64.3% to 70% [26]. SARC-Calf is composed of six items: the same five items as the original SARC-F (scored the same), with the sixth item being CC. The CC item score is 0 points when its value is above the cut-off value, and it can take a maximum value of 10 points when its value is below or equal to the cut-off point. A score ≥11 reveals a positive screening for sarcopenia [27]. Therefore, SARC-CalF can be used as a quick and better screening tool for sarcopenia in primary care [25].

The Mini Sarcopenia Risk Assessment Questionnaire (MSRA) is another promising sarcopenia screening tool. It is available in two versions: (a) the short one with five items (MSRA-5) and (b) the full one with seven items (MSRA-7) [28]. The MSRA-7 questionnaire examines (1) age, (2) recent hospitalization, (3) physical activity level, (4) frequency of meals, (5) dairy intake, (6) protein intake and (7) unintended loss of body weight (>2 kg in the last year). MSRA-5 does not include the intake of dairy products and proteins. A risk of sarcopenia is indicated by a total score of ≤30 and ≤45 points for MSRA-7 and MSRA-5, respectively. MRSA-5 may be used as a more effective screening tool for detecting sarcopenia in Chinese community dwellings compared to MSRA-7, exhibiting a significantly higher diagnostic accuracy (85% vs. 70%; *p* < 0.001) [29]. In the same study, SARC-F demonstrated a higher diagnostic accuracy than that of MSRA-5 (89% and 85%, respectively; *p* = 0.130), as well as greater specificity (98.1% vs. 70.6%, respectively). However, MSRA-5 has better sensitivity compared to SARC-F (90.2% vs. 29.5%, respectively) [29]. Nonetheless, further studies are necessary to verify the diagnostic value of these questionnaires.

### 3.2. Definition and Diagnostic Criteria of Sarcopenia

Various scientific groups, including the EWGSOP, the IWGS, the AWGS, the American Foundation for the National Institutes of Health (FNIH) and the Sarcopenia Definition and Outcomes Consortium (SDOC), have published consensus papers outlining diverse definitions of sarcopenia. The definitions and criteria of sarcopenia are described in Table 1, and the recommended cut-off points are shown in Table 2.

### 3.3. Formatting of Mathematical Components

In 2010, the EWGSOP introduced a clinical definition of sarcopenia and recommended cut-off points for assessing muscle mass, muscle strength and physical performance [30]. Over the past decade, these guidelines have contributed significantly to shaping the criteria for sarcopenia. Therefore, the initial definition of sarcopenia by the EWGSOP outlined three terms:

(a) Pre-sarcopenia, the presence of low muscle mass;
(b) Sarcopenia, the presence of low muscle mass with low muscle strength or poor physical performance;
(c) Severe sarcopenia, the presence of low muscle mass, low muscle strength and poor physical performance [30].

In 2019, the European Working Group modified their first definition of sarcopenia in order to reflect novel and updated scientific and clinical information that had been established over the last decade [9]. The updated definition of sarcopenia by the European group (EWGSOP2) is characterized as follows:

(a) Probable sarcopenia, the presence of low muscle strength;
(b) Confirmed sarcopenia, the presence of low muscle quality and quantity;
(c) Severe sarcopenia, the co-existence of poor physical performance together with low muscle strength and low muscle quality/quantity [9].

The cut-off points are determined by the measurement tool and the availability of relevant research data. The first EWGSOP guidelines did not refer to exact cut-off points, but they used cut-off points at two standard deviations below the mean reference value of healthy young adults [30]. The EWGSOP2 recommends simple and specific cut-off values to determine sarcopenia. In the updated criteria, the cut-off points for handgrip strength in both males and females are lower than the first recommendation, and they are close to the cut-offs proposed by all the other scientific working groups [9]. The prevalence of sarcopenia is underestimated using the handgrip strength cut-off values of the EWGSOP2 [33]. As a result, using the EWGSOP2 cut-off points could lead to underestimation of the actual sarcopenia phenotype.

On the contrary, the IWGS defined sarcopenia as low muscle mass in addition to poor physical performance, without considering muscle strength. The IWGS criteria of sarcopenia incorporate low muscle mass and low physical performance as key parameters. According to this group, sarcopenia should be emphasized in bedridden patients who are unable to independently rise from a chair or who have a slow gait speed. Individuals who meet these criteria need more careful and strict body composition assessments [31]. The IWGS also provides cut-off points for gait speed and muscle mass.

In 2014, the Asian Working Group (AWGS) suggested that low muscle mass, low muscle strength and/or poor physical performance define sarcopenia [10]. In 2019, the Asian Working Group revised their initial diagnostic criteria of sarcopenia by changing algorithms and cut-off values [14]. The AWGS2 proposed separate algorithms for community and clinical settings and introduced the term “possible sarcopenia”, defined by low muscle strength with or without low physical performance [14]. However, the definition of possible sarcopenia is recommended for patients living in a community but not in clinical or research settings. Both the initial and updated AWGS criteria define lower cut-off points for muscle mass, muscle strength and physical performance than those of the EWGSOP and IWGS [10,14,30,31]. According to the AWGS, DXA is the most widely used technology for assessing muscle mass in sarcopenia research, whereas BIA is suggested for community-based screening programs due to its accessibility, low cost, quick processing, noninvasiveness, radiation-free features and simplicity [10]. However, the cut-off values of sarcopenia using DXA in Asian populations are lower than those in Caucasian populations, as recommended by the EWGSOP and IWGS. The lower cut-off points are attributed to Asians’ lower body weight compared to that of Caucasian populations. Thus, the prevalence of sarcopenia is relatively low in Asian studies. Furthermore, there are significant differences in socioeconomic circumstances, such as culture and lifestyle, between the two races [34]. In conclusion, it is more appropriate to use the respective cut-off points for Asians and Caucasians to define low muscle strength and low muscle mass, since (a) Asians have lower values of body weight and handgrip strength, (b) there is a lower prevalence of sarcopenia in Asians and (c) different lifestyles are present between Asians and Caucasians of European descent [10].

The recommendations of the FNΙH are similar to those of the EWGSOP, suggesting the assessment of physical performance through gait speed first, followed by an assessment of handgrip strength and an assessment of lean mass with DXA [32]. The main difference in the FNIH criteria from the EWGSOP, EWGSOP2, AWGS and IWGS criteria is that that the FNIH suggested a new muscle mass index adjusted for BMI (ALM/BMI) instead of a muscle mass index adjusted for height (ASM/height^2^) [9,10,30,31,35].

In 2020, the SDOC published a different definition of sarcopenia. Sarcopenia was defined as the presence of both muscle weakness and slowness. Τhe SDOC suggested sex-specific cut-off points for muscle weakness (low handgrip strength) and slowness (slow gait speed) [13]. These criteria were used because low muscle strength and low physical performance can predict disability, falls, hip fractures and mortality. The main difference between the SDOC and previous definitions lies in the fact that lean body mass (LBM) is not used as a criterion. Slow gait speed was also found to be the best predictor of slowness in community-dwelling elders [13]. The cut-off values for handgrip strength proposed by the SDOC are higher compared to those of the other scientific groups, and they are also higher than the initial recommendations by the EWGSOP criteria (for example, the handgrip strength value for men defining sarcopenia is <35.5 kg according to the SDOC and <30 kg according to the EWGSOP) [13,30]. This can be explained by noting that the SDOC criteria do not take muscle mass into account. Thus, higher cut-off points for both men and women are recommended to classify more adults as sarcopenic, as they have muscle weakness [35].

In conclusion, low muscle mass is recommended as a diagnostic criterion for sarcopenia by all working groups, except for the SDOC. Therefore, more studies are needed to determine whether all three criteria (low muscle mass, low muscle strength and poor physical activity) or only two of them (according to the SDOC or IWGS) are required to improve diagnostic validity for sarcopenia.

### 3.4. Muscle Mass Assessment

A variety of radiological imaging techniques, including DXA, MRI and CT, can be utilized for the clinical diagnosis of sarcopenia [36]. These methods assess both muscular mass and quality, offering valuable information.

DXA is used widely as a radiological technology for determining body composition [37] in both clinical and research settings due to its affordability, accuracy and acceptance among the elderly [38]. DXA can effectively measure three body composition compartments: lean mass (LM), fat mass (FM) and bone mineral density (BMD), offering the advantage of regional estimates for each component [36]. Notably, DXA is characterized by its quick measurement speed (<20 min for a whole-body scan) and cost effectiveness [37]. However, a key limitation of DXA is its inability to assess the quality of muscle mass, as it cannot detect adipose tissue within the muscles [36,37]. Additionally, measurements may be influenced by hydration status [36]. The non-portability of DXA equipment further restricts its use among community-dwelling individuals [30,39]. Another drawback is the significant variability in analytical methods between different manufacturers and models, limiting the comparability of measurements obtained from different scanners [36,37].

The gold standard approaches for evaluating body composition are MRI and CT scans [38]. Both imaging techniques are capable of distinguishing fat from other soft tissues in the body [30] and concurrently quantifying both muscle quantity and quality [37]. However, a notable drawback of CT is the high radiation exposure, limiting its clinical utility for routine body composition assessments [5]. Despite this limitation, CT remains a suitable imaging technique for effectively evaluating sarcopenia in the screening and follow-up of various degenerative diseases, such as cancer [36]. Furthermore, both MRI and CT have shared limitations, including (a) a high cost, (b) lack of portability, (c) space requirements and (d) the need for highly trained technical experts [37]. Additionally, consistent establishment of cut-off points for low muscle mass has yet to be achieved for these imaging techniques [9].

Ultrasound has recently gained popularity in clinical and research settings due to its cost-effectiveness, widespread availability and non-ionizing radiation exposure [40]. Ultrasound facilitates the easy measurement of muscle quantity and quality, allowing for the identification of muscle wasting over time [9]. This method has demonstrated good validity in assessing muscle mass when compared to other imaging techniques such as DXA, CT and MRI [41]. Although the EWGSOP2 criteria suggest ultrasound as a valid method for assessing muscle mass, no specific cut-off values are provided [9]. Conversely, the AWGS 2 does not recommend the use of ultrasonography to estimate muscle mass due to a lack of supporting studies [14]. Further research is required to validate predictive equations for assessing muscle mass in patients with sarcopenia, both with and without comorbidities.

BIA is a cost-effective, simple-to-use, repeatable and versatile method suitable for both mobile and bedridden individuals [9]. However, it does not directly measure muscle fat and muscle mass; instead, it primarily estimates these components based on the differential conduction of current through tissues [37]. This method can estimate the volume of lean mass and body fat but cannot directly measure the ASM [10]. The precision of BIA measurements is subject to various factors, including age, ethnicity, hydration status, medical condition and comorbidities, recent food intake and exercise [42]. Due to the availability of various brand instruments for muscle mass estimation, the EWGSOP2 suggests using the raw measurements produced by each device, also using the proposed equipment equation. In addition, for standardization purposes, it is appropriate to incorporate the cross-validated Sergi equation [9,43]. The InBody, TANITA-MC, BIA 101 (Akern) and Bodystat devices are most commonly used in research and clinical settings to measure ASM, which also has validated equations [14]. Notably, BIA may overestimate muscle mass and underestimate fat mass [44]. Nevertheless, BIA can serve as a portable alternative to DXA, as it has been recognized as a valid method by both European and Asian guidelines for assessing sarcopenia [9,10].

The phase angle, directly derived from various Bioelectrical Impedance Analysis (BIA) instruments, serves as a valuable measure for evaluating both muscle quantity and quality, as well as physical function [45,46,47,48]. The EWGSOP2 criteria acknowledge the phase angle as a robust marker for assessing muscle mass [9]. However, it is important to note that specific cut-off values for the phase angle in the context of muscle mass assessment are not provided by the EWGSOP2 criteria. Several reference values have been provided in populations [49,50,51,52]. Nevertheless, the phase angle derived from BIA instruments has been recognized as a useful metric in evaluating muscle health and functionality. In addition, standardized phase angle values may prove to be a useful tool in the future [53], although specific cut-offs for the standardized values of the phase angle are still lacking. It should be noted that the type of machine [54] as well as the measuring position (standing vs. supine) [55] may differentiate deriving values for the phase angle, suggesting that, at best, the reference values used should be measured with the same machine. Physiological factors [56,57], diet [58,59] and diseases [47] may also affect phase angle values.

In conclusion, DXA is the predominant imaging technique for identifying sarcopenia, offering established cut-off points and low radiation exposure [36]. The clinical applications of DXA are widespread, mainly in primary health, geriatric medicine and metabolic diseases. On the other hand, CT and MRI find more prevalent use in diagnosing and monitoring chronic diseases [5]. Additionally, DXA is favored over BIA due to its direct measurement of body composition, whereas BIA relies on specific prediction equations to estimate muscle mass rather than directly measuring it [60].

### 3.5. Muscle Strength Assessment

There are limited validated methods for measuring muscle strength, with handgrip strength being widely employed. Additionally, tests such as the knee extensor test and chair stand test have been suggested as measurements of lower limb strength.

Handgrip strength has emerged as the most extensively utilized method, serving as a robust indicator of compromised physical mobility compared to low muscle mass. Moreover, the handgrip strength test is notably straightforward, portable, quick and cost-effective [61]. Handgrip strength is moderately related to strength in various body regions, so it is used as a reliable proxy for more complex measurements of both arm and leg strength [10]. It is worth noting that diverse methods exist for measuring handgrip strength, encompassing equipment and measurement protocols, leading to variation across studies. Among the accepted assessment tools, the hand dynamometer is commonly employed to measure handgrip strength [62]. The Jamar dynamometer is recognized as the gold standard method and is frequently reported as the primary tool for measuring handgrip strength [63].

However, caution is essential when relying on a single method to assess overall muscle strength, given the diversity of activities that involve both upper and lower body function [64]. The knee extensor muscle strength test has proven to be crucial in various functional tasks, including walking, rising from a chair and climbing stairs [65]. Notably, knee extensor strength tends to decline more quickly with aging than handgrip strength, making the knee extensor strength test valuable for sarcopenia diagnosis [66]. Furthermore, knee extensor strength is considered a better prognostic factor for functional performance than grip strength in older individuals living in assisted living facilities, compared to those in community-dwelling settings and nursing home residents [67].

The chair stand test, as recommended by the updated guidelines by the European Working Group (EWGSOP2), serves as a valuable proxy method for assessing lower strength. It has demonstrated efficacy as a straightforward screening tool for identifying sarcopenia in older adult females [68]. In this test, participants are timed as they transition from a seated to a standing position without utilizing their arms and then return to the chair, repeated five times [68]. Notably, there is evidence suggesting that the chair stand test is not directly linked to muscle mass. However, the test has proven effective in gauging overall physical performance and exhibits a correlation with a lower prevalence of sarcopenia when compared to the grip strength test [69].

### 3.6. Physical Performance Assessment

There are several tests that can assess physical performance, with the most common being the Short Physical Performance Battery (SPPB), usual gait speed and the Time Up and Go test (TUG).

The SPPB has emerged as a highly promising tool for evaluating functional ability and determining the biological age of older adults. This objective assessment focuses on the physical performance of the lower extremities. The SPPB comprises three timed tasks: (a) the chair stand test (as described earlier), (b) usual gait speed and (c) standing balance. In the gait speed test, participants are instructed to walk 4 m at their usual pace. For the standing balance test, participants are initially required to stand with their feet side by side for about 10 s. Subsequently, they must place the instep of one foot touching the big toe of the other foot for approximately 10 s [70]. The score ranges from 0 to 12 points, reflecting minimum to maximum performance [70], and a score <8 points indicates low physical performance [9]. The completion time for the SPPB is typically around 10–15 min [70]. Widely utilized in clinical practice and research, the SPPB serves as a comprehensive composite test for assessing physical performance, according to the EWGSOP criteria [30].

Although usual gait speed is an integral component of the SPPB, it can also be used as a standalone test in both research and clinical settings [30]. Serving as a straightforward, cost-effective and accurate measure of functional ability, gait speed has demonstrated its predictive value for significant health-related outcomes [71]. Notably, the test requires no specialized equipment, only a flat floor without barriers [8]. As mentioned before, participants are instructed to walk 4 m at their typical pace [72]. A gait speed below 0.8 m/s is widely recommended as the cut-off value indicating poor physical function, applicable to both men and women [9,10].

The TUG test assesses the time taken to perform a series of functionally relevant tasks. This includes rising from a chair, walking 3 m at the participant’s usual gait, turning, walking back and sitting down. Widely utilized in clinical settings, the TUG test serves as a valuable tool for evaluating gait, dynamic balance and fall risk [73]. The cut-off values for identifying individuals at high risk of falling can vary based on the characteristics of the studied population. In clinical settings, an individual who takes ≥13.5 s to complete the TUG test is considered at risk for falling [74].

Various tests, including the 400 m walk test, 6 min meter walking test and stair climb power test, can be employed to evaluate physical function mainly in research settings [8,30,75]. Nevertheless, gait speed stands out as the most frequently utilized tool in clinical settings due to its appropriateness, cost-effectiveness, speed and reliability in assessing overall mobility [76].

### 3.7. Biomarkers

Sarcopenia is characterized by a multifactorial pathogenesis, involving (a) age-related changes in hormone levels and neuromuscular sensitivity and (b) age-related decline in muscle mass due to a chronic pro-inflammatory state and oxidative stress. Developing a single validated biomarker could be a simple and cost-efficient method for diagnosing and monitoring sarcopenic patients [9]. However, it is preferable to establish a panel of complementary markers, including imaging techniques, serum biomarkers and functional tests, to collectively provide an optimal biomarker panel for diagnosing sarcopenia [77]. Inflammatory markers, such as tumor necrosis factor (TNF) and interleukin-6 (IL-6), as well as testosterone and growth hormones, have been identified for sarcopenia diagnosis [78,79]. Additionally, proposed diagnostic markers for sarcopenia include visceral proteins, 3-Methylhistidine, creatinine and carnitine [80]. Given the complex and multifactorial nature of sarcopenia, it is essential to identify a panel of serum biomarkers for both disease diagnosis and treatment efficacy. However, further studies are strongly recommended to establish an efficient biomarker panel applicable in clinical practice.

Genome-wide association studies have also identified genomic risk loci linked to sarcopenia features, including low grip strength and muscle mass. In a genome-wide meta-analysis, three SNPs were linked to low grip strength, with variants rs34415150 of HLA-DQA1, rs143384 of GDF5 and rs62102286 of DYM showing the strongest associations. Another SNP, rs10952289 near the AOC1 gene, was found to be associated with ALMM [81]. Additionally, in patients with sarcopenia, MT1X and ARHGAP36 were upregulated, whereas FAM171A1, GPCPD1, ZNF415 and RXRG, were downregulated, demonstrating high diagnostic accuracy for sarcopenia and potential as predictive markers for screening [82]. In silico analysis also revealed 78 SNPs related to sarcopenia, associated with falls and various lifestyle factors, such as smoking, alcohol consumption, nutritional habits (processed meat, salt and bread intake) and a sedentary lifestyle. These genes are expressed in diverse tissues, including adipose tissue, skeletal muscle, the thyroid, arteries, and the nervous system [83]. Circulating microRNAs that are related to sarcopenia have been identified. Increased expression of microRNA-1, microRNA-29a and microRNA-29b and decreased expression of microRNA-486, microRNA-146a, microRNA-206, microRNA-133a, microRNA-133b, microRNA-208b and microRNA-499 were observed in patients with low physical performance [84]. In a study with sarcopenic patients, microRNA-155, microRNA-208b, microRNA-222, microRNA-210, microRNA-328 and microRNA-499 were downregulated compared with non-sarcopenic patients. Furthermore, microRNA-208b and microRNA-155 correlated with handgrip strength in women, and microRNA-208b, microRNA-499 and microRNA-222 correlated with ASM/Height^2^ in men [85]. These genetic loci offer potential as markers for early diagnosis and treatment of sarcopenia.

## 4. Comparative Examination of Diagnostic Criteria

We searched Pubmed for observational studies comparing the diagnostic criteria of sarcopenia, as outlined in Table 3. Nine studies were retrieved that fulfilled the inclusion criteria.

The vast majority of the included studies focused on elderly subjects (>60 years), with the exception of the studies of Spextoto (>50 years) [86] and Pang et al. (21–90 years) [87]. Across all studies, muscle strength was assessed using a dynamometer. Muscle mass assessment was predominantly performed with BIA [88,89] and DXA [87,90,91,92,93], and few studies utilized predictive equations and anthropometric measurements [86,94]. In the assessment of physical performance, high variability was observed.

Several studies have compared sarcopenia diagnosis with the EWGSOP and EWGSOP2 criteria [86,88,89,90,91,94]. The prevalence of sarcopenia with the EWGSOP2 criteria has been found to be both lower [88,91] or higher [90] than that resulting from the use of the EWGSOP criteria. However, the EWGSOP2 criteria seem to be more sensitive [89] and to have a better predictive value against mortality [86].

Comparisons of the EWGSOP2 with FNIH criteria have been conducted in the studies of Yang et al. [88] and Sim et al. [93]. The prevalence of sarcopenia was higher when the EWGSOP criteria were used. Fall-related hospitalization risk was not related to sarcopenia with all criteria used [93]. Less data are available regarding the performance of the EWGSOP criteria versus the IWGS [92]. In this study, differences were observed in sarcopenia diagnosis, but no consistent differences have been reported [92]. Similarly, two studies compared the EWGSOP to the AWGS criteria [87,88]. The prevalence of sarcopenia was lower when the EWGSOP criteria were used [88]. In another study, sarcopenia prevalence according to the EWGSOP2 was lower than that according to the AWGS2019 criteria but was relatively close to that according to the AWGS2014 criteria [87]. It is noted that sarcopenia prevalence in the study of Pang et al. increased from 6.7% to 13.6% when the AWGS2014 and AWGS2019 criteria were used, respectively [87]. Interestingly, the study of Pang et al. revealed that, among younger and middle-aged adults (21–59 years), 6.9% had confirmed sarcopenia, suggesting that sarcopenia is not only a phenomenon of the elderly [87].

## 5. Public Health Solutions

The impact of nutrition on the maintenance of muscle mass and its protective effects against sarcopenia underscore the crucial role of adequate protein consumption. The ESPEN recommends a minimum protein intake of 1.0 g/kg/BW for older adults, considering factors such as nutritional status, physical activity levels and comorbidities [95]. Numerous randomized controlled trials (RCTs) have explored the type and amount of protein intake to prevent and treat sarcopenia. Leucine [96], the co-administration of beta-methyl butyric acid (HMB), arginine, glutamine [97] and casein [98] have demonstrated a protective role in preserving muscle mass. Creatine (Cr) supplementation can also be used accompanied with exercise to enhance muscle regenerative capacity [99,100]. Supplementation with creatine monohydrate (CrM) leads to an approximate 25% increase in total creatine levels in skeletal muscle. However, when combined with exercise training, the elevation can reach up to 37% [101,102]. Evidence about the impact of vitamins in muscle mass and strength is limited and controversial. Studies on vitamin D supplementation suggest its potential to increase muscle mass and strength [103,104]. Vitamin D deficiency, conversely, has been linked to reduced skeletal muscle mass [105], impaired physical function [106] and the onset of sarcopenia. Genetic factors, such as the FOK1 CG genotype associated with sarcopenia and the impact of FOK1 Bsm1 SNPs on muscle mass and muscle strength in CG and CT, further contribute to this association [107]. Although studies on antioxidants [108], *n*-3 fatty acids [109] and vitamin K [110] suggest links with muscle traits and sarcopenia, the evidence remains limited. Inadequate nutrient consumption, including a particularly low protein intake, may lead to malnutrition in older adults, accelerating the onset of sarcopenia. Therefore, implementing appropriate nutritional interventions is paramount in both preventing and treating sarcopenia.

Exercise plays a pivotal role in addressing and mitigating sarcopenia. Regular physical activity, particularly a combination of resistance training [111,112] and aerobic exercise [113], has been shown to be effective in preventing and treating this condition. Progressive resistance training enhances muscle cross-sectional area and improves handgrip strength and physical function in older adults [112,114]. High-intensity interval training (HIIT) can also improve overall sarcopenia in elders, enhancing functional capacity and muscle mass [115,116]. Therefore, implementing appropriate nutritional interventions in combination with exercise, especially resistance exercise, is paramount in both preventing and treating sarcopenia.

## 6. Conclusions

Sarcopenia constitutes a significant geriatric issue often manifesting from early middle age. Its diagnosis remains a crucial and controversial matter due to the existing diverse diagnostic criteria. The common denominator among the diagnostic criteria is the parameters evaluated for diagnosis, which include low muscle mass, low muscle strength and physical fitness. The disparity between existing diagnostic criteria lies in the cut-off thresholds for the measurements of each parameter. The selection of appropriate diagnostic criteria depends on nationality and the feasibility of measurements in both clinical and research settings. It is recommended to measure all three parameters of sarcopenia, utilizing suitable tools and methods, always considering the available equipment and qualified personnel. In addition, the evaluation of more novel parameters, such as the phase angle and comparability to existing criteria, is of additive value.

## Figures and Tables

**Table 1:** Diagnosis of sarcopenia according to international working groups.

Consensus Group	Year	Criteria for Diagnosis	Notes
EWGSOP [30]	2010	Low muscle mass Low muscle strength Low physical performance	Sarcopenia is classified according to the criteria as follows: Pre-sarcopenia, when only low muscle mass exists Sarcopenia, when low muscle mass with low muscle strength or physical performance exists Severe sarcopenia, when all three criteria co-exist.
IWGS [31]	2011	Low muscle mass Low physical performance	Older adults with both low muscle mass and function should be considered patients with sarcopenia.
FNIH [32]	2014	Low muscle mass Low muscle strength Low physical performance	Based on a thorough examination of clinically relevant thresholds for weakness and low LBM.
AWGS [10]	2014	Low muscle mass Low muscle strength Low physical performance	Same as the EWGSOP definition Cut-off points are used that are specific to elderly Asian people or those who are descended from Asians.
EWGSOP2 [9]	2019	Low muscle mass Low muscle quantity or quality Low physical performance	Updated definition of sarcopenia Sarcopenia is classified according to the following criteria: Probable sarcopenia, when low muscle strength exists Sarcopenia, when low muscle strength and low muscle quantity and/or quality exist Severe sarcopenia, when all criteria co-exist.
AWGS 2 [14]	2020	Low muscle strength Low muscle mass Low physical performance	Sarcopenia is classified according to the following criteria: Possible sarcopenia, when low muscle strength with or without low physical performance exist Sarcopenia, when low muscle mass, low muscle strength and/or low physical performance exist Severe sarcopenia, when all criteria co-exist.
SDOC [13]	2020	Low muscle strength Low physical performance	The definition of sarcopenia is the existence of both slowness and muscle weakness, regardless of lean mass measured by DXA. Low DXA-derived LBM has no consistent connection with negative health consequences (falls, mobility, and mortality).

EWGSOP = European Working Group on Sarcopenia in Older People; IWGS = International Working Group on Sarcopenia; FNIH = American Foundation for the National Institutes of Health; LBM = Lean Body Mass; AWGS = Asian Working Group for Sarcopenia; SDOC = Sarcopenia Definition and Outcomes Consortium; DXA = Dual-energy X-ray absorptiometry.

**Table 2:** Methods and cut-off points for diagnostic criteria by different working groups.

	EWGSOP [30]	IWGS [31]	FNIH [32]	AWGS [10]	EWGSOP2 [9]	AWGS 2 [14]	SDOC [13]
Muscle Mass	DXA (ALM/height^2^): <7.26 kg/m^2^ ♂ <5.5 kg/m^2^ ♀ or BIA: <8.87 kg/m^2^ ♂ <6.42 kg/m^2^ ♀ or CT or MRI or Total or partial body potassium per fat-free soft tissue	DXA (ALM/height^2^): <7.23 kg/m^2^ ♂ <5.67 kg/m^2^ ♀	DXA (ALM/BMI): <0.789 kg/BMI ♂ <0.512 kg/BMI ♀	DXA (ALM/height^2^): <7.0 kg/m^2^ ♂ <5.4 kg/m^2^ ♀ or BIA: ≤7.0 kg*m^−2^ ♂ <5.7 kg*m^−2^ ♀	DXA (ALM/height^2^): <7.00 kg/m^2^ ♂ <6.00 kg/m^2^ ♀ or BIA or CT or MRI	DXA (ASM): <7.0 kg/m^2^ ♂ <5.4 kg/m^2^ ♀ or BIA (ASM): ≤7.0 kg*m^−2^ ♂ <5.7 kg*m^−2^ ♀	Not specified
Muscle Strength	Grip strength: <30 kg ♂ <20 kg ♀ or Knee flexion/extension or Peak expiratory flow	Not specified	Grip strength: <26 kg ♂ <16 kg ♀	Grip strength: <26 kg ♂ <18 kg ♀	Grip strength: <27 kg ♂ <16 kg ♀ or Chair stand test >15 s	Grip strength: <28 kg ♂ <18 kg ♀	Grip strength: <35.5 kg ♂ <20 kg ♀
Physical Performance	SPPB: <8 or 4MGS: <0.8 m/s or TUG or SCTP	4MGS: <1.0 m/s or Standing up from a chair	4MGS, 6MGS: <0.8 m/s	6MGS: <0.8 m/s	Gait speed: <0.8 m/s or SPPB: ≤8 or TUG: ≥20 s or 400 m walk: >6 min	6MGS: <1.0 m/s or 5TSST: >12 s or SPPB: ≤9	Gait speed: <0.8 m/s

EWGSOP = European Working Group on Sarcopenia in Older People; IWGS = International Working Group on Sarcopenia; FNIH = American Foundation for the National Institutes of Health; AWGS = Asian Working Group for Sarcopenia; SDOC = Sarcopenia Definition and Outcomes Consortium; DXA = Dual-energy X-ray absorptiometry; ALM = Appendicular lean mass; kg = Kilogram; m = Meter; BIA = Bioimpedance analysis; CT = Computed tomography; MRI = Magnetic resonance imaging; BMI = Body mass index; ASM = Appendicular skeletal mass; s = Second; SPPB = Short Physical Performance Battery; 4MGS = 4 m gait speed; TUG = Time Up and Go; SCTP = Stair climb power test; 6MGS = 6 m gait speed; 5TSST = 5 times stand-to-sit test. ♂ = male; ♀ = female.

**Table 3:** Results of studies comparing the diagnostic criteria for sarcopenia.

Author	*n*	Study Population	Diagnostic Criteria	Muscle Mass	Muscle Strength	Physical Performance	Results
Spexoto et al., 2022 [86]	6.182	≥50 year-old community-dwelling individuals living in England	EWGSOP and EWGSOP2	ASMM (kg/m^2^) determined by using the Lee equation	Handgrip strength was measured by using a dynamometer with 3 trials on the dominant hand. The best performance was recorded.	2.4 m gait speed; 2 trials; The best performance was recorded.	The EWGSOP2 was a better predictor of mortality risk than the EWGSOP.
Pang et al., 2021 [87]	542	≥60 year-old community-dwelling individuals living in Singapore	AWGS, AWG2 and EWGSOP2	DXA (ALMI, ALM/height^2^)	Handgrip strength was measured by using a dynamometer with 2 trials per arm. The best performance was recorded.	6-m gait speed; 3 trials; The average was recorded.	According to the AWGS 2, the prevalence of sarcopenia was greater compared to the AWGS and EWGSOP2 criteria in participants aged ≥60 years.
Yang et al., 2020 [88]	483	≥60 year-old Chinese community-dwelling individuals	EWGSOP, EWGSOP2, AWGS, IWGS and FNIH	ΒΙA (SMI, ASM/BMI)	Handgrip strength was measured by using a dynamometer with 3 trials for each hand. The best performance for each hand was recorded.	4 m gait speed; 2 trials; The best performance was recorded.	The prevalence of sarcopenia as defined by the EWGSOP2 (men: 6.5%; women: 3.3%) was lower than those defined by the EWGSOP (men: 22.3%; women 11.7%), AWGS (men: 10.9%; women: 8.0%) and IWGS (men: 24.5%; women: 11.0%) criteria but higher than the FNIH criteria (men: 6.0%; women: 1.7%).
Yang et al., 2019 [89]	384	≥60 year-old community-dwelling individuals living in China	EWGSOP and EWGSOP2	BIA (ASMI, ASM/height^2^)	Handgrip strength was measured by using a dynamometer with 3 trials per arm. The best performance was recorded.	4 m gait speed	The EWGSOP2 defined a lower prevalence of sarcopenia than that of the EWGSOP criteria.
Wallengren et al., 2021 [90]	1.041	One cohort included ≥70 year-old participants, and the second cohort included ≥85 year-old participants from Sweeden	EWGSOP and EWGSOP2	DXA (ALSTI kg/m^2^)	Handgrip strength was measured by using a dynamometer with 3 trials per arm. The best performance was recorded.	30 m gait speed	A 0.9–1.0% lower prevalence of sarcopenia was determined by using the EWGSOP2 compared to the EWGSOP 1 (*p* < 0.005).
Shafiee et al., 2020 [91]	2.426	≥60 year-old community-dwelling individuals from Iran	EGWSOP and EWGSOP2	DXA (SMI kg/m^2^)	Handgrip strength was measured by using a dynamometer with 3 trials per arm. The best performance was recorded.	4.57 m gait speed	EWGSOP2 defined a lower prevalence of sarcopenia than that of the EWGSOP criteria for both males and females.
Lee et al., 2013 [92]	408	≥50 year-old community-dwelling individuals living in Taiwan	IWGS and EWGSOP	DXA (RASM, SMI %)	Handgrip strength was measured by using a dynamometer with 3 trials on the dominant hand. The best performance was recorded.	6 m gait speed; 2 trials; The shortest time was recorded.	The EWGSOP criteria defined a significantly higher prevalence of sarcopenia (7.8% vs. 4.1%, *p* < 0.001 by RASM, and 16.6% vs. 11.1%, *p* < 0.001 by SMI) compared to the IWGS.
Sim et al., 2019 [93]	903	≥70 year-old community-dwelling Caucasian-Australian women	FNIH, EWGSOP and modified FNIH (AUS-POP_F_) and EWGSOP (AUS-POP_E_)	DXA (ALM/BMI and ALM/height^2^)	Handgrip strength was measured by using a dynamometer with 3 trials on dominant hand. The best performance was recorded.	TUG	Both the FNIH and EWGSOP sarcopenia definitions were predictive of future fall-related risk.
Bachettini et al., 2020 [94]	1.291	≥60 year-old community-dwelling individuals from Brazil	EWGSOP and EWGSOP2	CC was measured using tape. Reduced muscle mass was determined if CC was ≤34 cm for men and ≤33 cm for women, according to cut-off values established from the same population.	Handgrip strength was measured by using manual digital dynamometers with 3 trials per arm. The best performance was recorded.	4 m gait speed; 2 trials; The best performance was recorded.	No statistically significant association between the diagnostic criteria of sarcopenia and mortality risk was found.

EWGSOP = European Working Group on Sarcopenia in Older People; EWGSOP2 = European Working Group on Sarcopenia in Older People 2; ASMM = Appendicular skeletal muscle mass; kg = Kilograms; m = Meter; AWGS = Asian Working Group for Sarcopenia; AWG2 = Asian Working Group for Sarcopenia 2; DXA = Dual-energy X-ray absorptiometry; ALMI = Appendicular lean mass index; ALM = Appendicular lean mass; IWGS = International Working Group on Sarcopenia; FNIH = American Foundation for the National Institutes of Health; ΒΙA = Bioimpedance analysis; SMI = Skeletal muscle index; ASM = Appendicular skeletal muscle; BMI = Body mass index; ASMI = Appendicular skeletal muscle index; ALSTI = Appendicular lean soft tissue index; RASM = relative appendicular skeletal muscle index; SMI % = percentage skeletal muscle index; AUS-POP_F_ = modified FNIH based on Australian population data; AUS-POP_E_ = Modified EWGSOP based on Australian population data; TUG = Time Up and Go; CC = Calf circumference; cm = Centimeter.
